# Adherence to 24-h movement guidelines among rural and regional children in Australia: an observational study

**DOI:** 10.1007/s00431-025-06444-7

**Published:** 2025-10-06

**Authors:** Claudia Strugnell, Cadeyrn J. Gaskin, Michelle Jackson, Jane Jacobs, Liliana Orellana, Andrew D. Brown, Colin Bell, Josh Hayward, Steven Allender, Simone J. J. M. Verswijveren

**Affiliations:** 1 Institute for Physical Activity and Nutrition, Deakin University, Geelong, Australia; 2 Global Centre for Preventative Health and Nutrition (GLOBE), Institute for Health Transformation, Deakin University, Geelong, Australia; 3 Biostatistics Unit, Faculty of Health, Deakin University, Geelong, Australia

**Keywords:** Accelerometry, Children, Movement guidelines, Physical activity, Rural, Sedentary behavior, Sleep

## Abstract

Adhering to 24-h movement guidelines protects children’s health and wellbeing. We investigated adherence among a sample of children in regional and rural Victoria, Australia. Analysis was conducted using baseline data from RESPOND, a large community-based obesity prevention intervention conducted in regional and rural Victoria, Australia. Children (aged approx. 9–12 years) self-reported screen time and wore a wrist-worn accelerometer for seven days to determine the mean daily time spent on moderate to vigorous physical activity (MVPA), light physical activity (LPA), sedentary and sleeping. Multi-level linear and logistic regressions were used to estimate associations between accelerometry outcomes and individual and school level demographics overall and by gender, accounting for school level clustering. Valid accelerometry data were obtained for 1,264 students. Twenty-two percent (22%) of boys and 16% of girls met all three movement guidelines and 11% boys and 9% of girls met none of the guidelines. Boys engaged in more MVPA, and less LPA than girls. Compared to those in grade 4 (aged approx. 9–10 years), students in grade 6 (aged approx. 11–12 years) had significantly reduced MVPA minutes (− 7.8; 95%CI –12.3, − 3.4); increased sedentary minutes (31.0; 95%CI 22.7, 39.3), and reduced odds of meeting screen time guidelines (odds ratio, 0.65; 95%CI 0.50, 0.84). Stratification by gender found these results to be consistent for boys and girls. Living in a medium or large rural town was associated with having 6.4 (95%CI 0.0, 12, 8) more minutes in MVPA (boys) and greater odds of adhering to screen time guidelines (OR, 1.96 (95%CI 1.02, 3.79) (girls) compared to living in regional centers. Sleep minutes were lower for students who spoke a language other than English at home (− 21.0 95%CI − 32.5, − 9.5). Only screen-time adherence for girls was associated with socioeconomic status.

*Conclusions*: This study highlights low adherence to three Australian movement behavior guidelines among this large sample of regional and rural Victorian children. Large gender-differences in duration and adherence to MVPA and screen-time guidelines and declines with increasing age (all guidelines), highlight the need for population-wide interventions.

**Table:** 

**What is Known:** • *Time spent in movement behaviors (moderate-to-vigorous physical activity, light-intensity physical activity, sedentary behavior and sleep are important for children’s health*. • *Few studies have examined device-measured movement behaviors and adherence to 24-h movement guidelines among regional children and whether this varies by gender, rurality and socioeconomic background*.	
**What is New:** • *This study found 22% of boys and 16% of girls met the 24-h movement recommendations, with 11% of boys and 9% of girls meeting no guideline*. • *Living in a medium or large rural town was associated with more moderate to vigorous physical activity in boys, and less screen-time among girls compared to those living in regional centers. Higher socioeconomic status was associated with less screen-time among girls*.

**Supplementary Information:**

The online version contains supplementary material available at 10.1007/s00431-025-06444-7.

## Background

Moderate-to-vigorous-intensity physical activity (MVPA), light-intensity physical activity (LPA), sedentary behavior, and sleep impact child health [1] and academic achievement [2]. The benefits of engaging in physical activity, limiting sedentary behavior, and obtaining sufficient sleep is reflected in the 24-h movement guidelines of several countries (e.g., Australia [3, 4], Canada [5], New Zealand [6], and Singapore [7]), and the Asia–Pacific region [8]). Children’s adherence to these guidelines has been associated with health-related quality of life (HRQoL), healthy diets, cognition, adiposity, fitness, cardiometabolic, mental, emotional, and social health [1]. Understanding the extent of population adherence to these guidelines is critical.

Multiple jurisdictions have developed 24-h movement guidelines, [3–8] Australian guidelines [3] stipulate children and young people (a) accumulate ≥ 60 min per day of MVPA consisting mainly of aerobic activities (e.g., running, bike riding, dancing), and (b) engage in several hours of LPA (e.g., walking, park play). Sedentary behavior guidelines stipulate children and young people (a) limit sedentary recreational screen time to ≤ 2 h per day, and (b) break up long periods of sitting. Sleep guidelines stipulate (a) that children aged 5–13 years obtain 9–11 h of uninterrupted sleep per night, and (b) have consistent bed and wake-up times.

Globally, few children meet the 24-h movement guidelines [9]. A meta-analysis of 76,928 6–12 year olds (18 studies published 2016–2021) found global 24-h movement guideline adherence was 10% (95% confidence interval [CI], 7.5%, 13.1%) [9], and lower in girls [6.9%; (95%CI 5.0%, 8.9%)] than boys [11.1%; (95%CI 7.8%, 14.3%)] [9]. In contrast, not meeting guidelines for any of physical activity, sedentary behavior, and/or sleep was 15.6% (95%CI 11.6%, 19.6%) with no gender difference [9]. A systematic review of 20 studies showed child self-report was the main form of data collection and those studies using objective device-based data typically had smaller samples [9]. Given the increased risk of bias in self-reported versus device-measured physical activity [10, 11], especially among children [12]; there is a need to conduct device-measured research for measuring child 24-h movement guideline adherence.

Australian guideline research has primarily focused on pre-school children [13–17] and adolescents [18–20], overlooking primary school-aged children [18, 21] using device-measured data [21]. Data from 1270 children (mean age 12 years) found adherence to all three guidelines was higher with self-report (30.3%) than when MVPA and sleep were device-measured and screen time self-reported (12.0%) [21]. Objective device-based data from larger, representative samples, especially among regional and rural school children and those from socioeconomically disadvantaged areas who experience higher levels of overweight and obesity compared to their metropolitan counterparts is needed [22]. A demographic in which little 24-h device-based and representative information exists [18]. Further, existing Australian data suggests regional children spend more time outside than those in cities and inner regional areas [23]; and time outdoors is associated with physical activity [24]. Less evidence exists about socioeconomic status and physical activity [25], sedentary behavior [26], or sleep duration [27, 28]. Thus, we anticipated adherence to be greater among boys than girls; given the evidence of multiple supportive factors increasing boys’ participation in physical activity more than girls’ [e.g., parental support for physical activity, and higher self-perceived competence in physical education [29].

Using 2019 device-based 24-h movement data from our Reflexive Evidence and Systems interventions to Prevention Obesity and Non-communicable Disease (RESPOND) trial [30], we aimed to:

1. Investigate adherence to the Australian 24-h movement guidelines among school-aged children, in the northeast region of Victoria, Australia.
2. Explore whether adherence to the 24-h guidelines differed by gender, rurality, socioeconomic status.

## Methods

### Design

Baseline data (collected March 27 to June 28 2019) from the RESPOND trial was analyzed [30].

### Ethics

This study was performed in line with the principles of the Declaration of Helsinki. Ethics approval for the trial was granted from the Deakin University’s Human Research Ethics Committee (2018–381) and the relevant education bodies (Victorian Government Department of Education and Training, 2019_003943; Catholic Archdiocese of Melbourne, Catholic Education Melbourne, 2019–0872; and Diocese of Sandhurst, 24^th^ May 2019). The trial was registered with Australian New Zealand Clinical Trials Registry (ACTRN12618001986268) (11/12/2018) [30].

### Setting

The trial included ten local government areas (LGAs) in the Goulburn Valley and Ovens Murry regions of northeast Victoria, Australia. These LGAs are classified as regional centers, large, medium or small rural towns according to the Modified Monash Model [31] discussed below. In 2016 the 10 LGAs had 19,124 children 5 = 12 years, with 8196 across grades 2, 4, and 6 [32].

### Recruitment of schools and students within schools

All 112 schools (including government, independent, and Catholic schools) within the 10 LGAs were invited to participate. All children attending these schools were eligible if they were in grades 2 (~ 7 to 8 years), 4 (~ 9 to 10 years), or 6 (~ 11 to 12 years). There were no exclusion criteria. Children were enrolled unless they returned a signed opt-out form from their parents or guardians; or verbally opted out. Due to a limited availability of accelerometers for the study, a sub-sample of children in grades 4 and 6 (but not those in grade 2) were asked to wear these devices. Approximately 50% of students in grades 4 and 6 were offered an accelerometer; within each eligible grade level all children in the first classroom group were offered to wear a device, followed by every alternating group in that year level (i.e., accelerometers were offered to students in the first classroom groups (e.g., 6 A, 4B) to attend data collection within grade 4 and grade 6). The analyses presented in this paper are from the data of children in grades 4 and 6.

### Measures

#### School characteristics

School characteristics were obtained from the Australian Curriculum, Assessment and Reporting Authority’s *My School* website [https://www.myschool.edu.au] [33], including school sector, geographic location, and socioeconomic status of the student population. School sector was government, independent, or Catholic (independent and Catholic were combined as ‘non-government’ for analysis). Rurality of the school postcode [31] was classified as Modified Monash (MM) 1 (major city), MM 2 (regional center) MM3 (large rural town) MM4 (medium rural town) MM5 (small rural town) MM6 (remote communities) or MM7 (very remote communities) (Supp Table 1). Socioeconomic status was determined using the Index of Community Socio-Educational Advantage (ICSEA), based on school (geographic location, proportion of indigenous students) and student factors (parents’ occupations, parents’ education) [34]. The median value of ICSEA is set at 1000, with higher values indicative of greater socio-educational advantage [34].

#### Demographic characteristics

Children self-reported demographic data, including date of birth (used to determine age), gender (boy, girl, prefer not to state), town/city and postcode of residence, country of birth, Aboriginal and/or Torres Strait Islander background, and language spoken at home which was categorized to whether they spoke English at home, or a language other than English (LOTE).

#### 24-h movement behaviors

Daily total MVPA, LPA, sedentary time (ST) and sleep were measured using tri-axial ActiGraph wGT3X-BT accelerometers (ActiGraph, Pensacola, Florida, USA) using a sampling frequency of 30 Hz and normal filter. Each child was instructed to wear an accelerometer on their non-dominant wrist for 7 consecutive days and to remove the devices only for water-based activities (e.g., bathing, swimming) and high contact sports (e.g., boxing, rugby, martial arts). ActiGraph data were downloaded using ActiLife v. 6.11.4, saved in raw format as.gt3x files for data processing.

ActiGraph files with raw accelerometer signals were processed using the open-source package GGIR [35, 36] in R [https://cran.r-project.org] and auto calibrated using local gravity as a reference [35]. The vector magnitude of acceleration corrected for gravity was calculated over 5-s epochs. Data were excluded from all analyses if post-calibration error was greater than 0.02 g [5] or fewer than 16 h of wear-time were recorded by either monitor during the 24 h day of interest [37]. Non-wear was estimated based on the standard deviation (at least 2 out of the 3 axes less than 13 mg) and value range of each axis (less than 50 mg), calculated for 60-min windows with 15-min moving increments [36]. Data were considered valid if participants provided at least three days of 16 h of wear time [38].

Total average ST (< 48.1 mg; inactivity), LPA (between 48.1 mg and < 201.4 mg), moderate-intensity (between 201.4 mg and < 707.0 mg) and vigorous-intensity physical activity were calculated using previously validated thresholds for this age group [39–41]. Average daily sleep was determined using Van Hees’ [42] validated nocturnal sleep detection algorithm. In brief, periods of sustained inactivity, defined as no changes in arm angle greater than 5 degrees for 5 min are classified as sleep.

A binary variable was created indicating whether a child met Australia’s 24-h the physical activity and sleep guidelines [3] using the *average across all days* method [43]. A child was deemed compliant with the physical activity guideline if average MVPA was ≥ 60 min/day across the days during for which valid physical activity was recorded. Similarly, sleep guideline compliant if average sleep time was ≥ 9 and ≤ 11 h/day over the days for which valid sleep was recorded.

Screen time was determined by the self-reported Core Indicators and Measures of Youth Health questionnaire [44]. Students were classified as guideline compliant if they reported ≤ 2 h of recreational screen time per day for at least 5 out of 7 days. We have previously published the reliability and validity coefficients of the MVPA and screen-time questionnaire components of the Core Indicators and Measures of Youth Health Survey in our protocol paper [30]. A supplementary analysis was also conducted including adherence to screen-time guidelines 7 days a week. Children were categorized as meeting 24-h movement guidelines if they simultaneously met the three components, MVPA, screen and sleep.

### Statistical analysis

Listwise deletion was used to exclude observations with missing data in required variable. Multi-level linear and logistic models were run to estimate the accelerometry (mins per day of activity) and binary (meeting guidelines) outcomes, including gender, year level, language other than English spoken at home, school rurality, school type and school socio-economic status, and including school as a random effect to adjust for clustering. Post-estimation margins were used to estimate mean or proportion of outcome for boys and girls, and the differences in each outcome between genders. Multi-level linear models were run by gender to estimate associations between the accelerometry outcomes (daily average min in each LPA, MVPA, inactivity, sleep) and demographic factors: year level (Grade 4/6), language other than English spoken at home (Yes/No), school rurality (Regional center (MM2), Large/medium rural town (MM3/4) or Small rural town (MM5), school type (government/non government) and school socioeconomic status (ICSEA: < 1000/≥ 1000)). Multi-level logistic models were fitted to estimate associations between binary outcomes (meeting guidelines for MVPA, sleep, and screen time 5 days a week) and the same independent variables as the linear regression. All models included each of the outlined demographic factors, and the percentage of accelerometer wear time as fixed effects; and school as a random effect to account for within-school clustering. Venn diagrams to estimate adherence to the 24-h movement guidelines were created in Stata using the user-written *venndiag* command. A p value of < 0.05 was considered significant for all analyses due to the exploratory nature of the analysis. All analyses were conducted in Stata version 18.0.

## Results

Of the 112 schools invited, 91 (81%) participated in the study. Across schools, 2,363 children in grades 4 and 6 were eligible for inclusion in the study, and 1,856 (78.5%) participated. 1,560 children, from 88 schools returned their monitors with data from 1,469 accelerometers able to be processed. Children were excluded due to; accelerometer inclusion criteria not being met (*n* = 136 children), demographic data missing (grade or gender missing, *n* = 4; preferred not to state gender, *n* = 1; language spoken at home missing, *n* = 11), data errors where total time > 24 h (*n* = 1) or accelerometer results were ± 3 SD from the mean (*n* = 52), leaving a sample of *N* = 1264 children from 84 schools (Supp Fig. 1). The analysis of the screen time guideline adherence had a further three students with missing data. Child and school characteristics are presented in Table 1. Most children were in grade 4 (55%) female (52%), from an English-speaking background (92.2%), and lived in small rural towns (70%), and attended government schools (94%) with ICSEA scores below the Australian median (64%).

**Table 1:** Description of participants

	Boys (*N* = 597)		Girls (*N* = 667)		Total *(N* = 1264)	
	*Mean*	*sd*	*Mean*	*sd*	*Mean*	*sd*
Mean (sd) age	10.7	1.1	10.7	1.1	10.7	1.2
	*n*	*Proportion*	*n*	*Proportion*	*n*	*Proportion*
Grade 4	342	27.1%	355	28.1%	697	55.1%
Grade 6	255	20.2%	312	24.7%	567	44.9%
LOTE at home	53	4.2%	46	3.6%	99	7.8%
**School level variables**						
	Participants			Schools		
	Boys (*n*)	Proportion	Girls (*n*)	Proportion	*n*	Proportion
ICSEA score < 1000	378	29.9%	423	33.5%	54	64.3%
**MMM rurality**						
Regional centers (MM2)	87	6.9%	103	8.1%	6	7.1%
Large/med rural town (MM3/4)	154	12.2%	234	18.5%	19	22.6%
Small rural town (MM5)	356	28.2%	330	26.1%	59	70.2%
**School type**						
Government	555	43.9%	607	48.0%	79	94.0%
Catholic	12	0.9%	30	2.4%	2	2.4%
Independent	30	2.4%	30	2.4%	3	3.6%

*LOTE* language other than English, *MMM* modified Monash model, *MM2* regional centers (pop 50,000), *MM3/4* large/medium town (pop 5000–50,000), *MM5* small rural town (pop < 5000), *ICSEA* index of community socio educational advantage

**Fig. 1:**
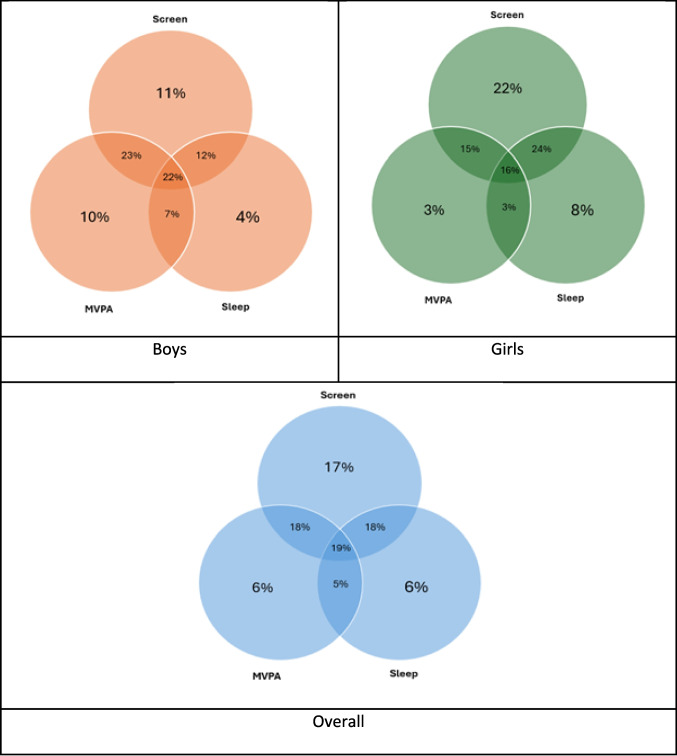
Venn diagram showing the proportion of boys, girls, and overall (%) meeting the physical activity (device measured), screen (self-report), and sleep guidelines (device measured). MVPA, moderate to vigorous physical activity

### Gender differences in physical activity, sedentary time and sleep duration and guideline adherence

Girls engaged in 13.5 min (95%CI, − 15.8, − 11.2,) less MVPA per day and 14.0 min (95%CI, 9.6, 18.4) more LPA per day than boys (Table 2). No gender differences in mean device measured sedentary time or sleep duration were observed, with participants typically engaging in 10 and half h and just under 9 h, respectively. Self-report data showed higher proportion of girls reporting that they meet the recommendation (difference, 9.8% (95%CI 4.7, 14.76)) compared to boys. Supplementary Fig. 2 also shows the average duration spent in each behavior on a typical 24-h day by gender.

**Table 2:** Mean minutes per day for physical activity, sedentary time and mean minutes per night for sleep, and proportion of participant meeting guidelines by gender

	Boys (*n* = 597)	Girls (*n* = 667)	Difference (girls-boys)
	Beta (95%CI)		
Valid wear days: weekdays	5.70 (5.6, 5.8)	5.8 (5.7, 6..00)	0.1 (0.0, 0.2)**
Valid wear days: weekend days	1.7 (1.6, 1.7)	1.7 (1.7, 1.8)	0.1 (0.0, 0.1)*
Mean LIGHT PA (mins)	214.3 (210.8, 217.8)	228.3 (224.9, 231.6)	14.0 (9.6, 18.4)**
Mean MPVA (mins)	68.1 (66.2, 69.9)	54.6 (52.8, 56.4)**	− 13.5 (− 15.8, − 11.2)**
Mean sleep (mins)	526.9 (522.4, 531.4)	530.8 (526.5, 535.0)	3.9 (− 2.4, 10.1)
Mean sedentary (mins)	631.2 (625.0, 637.3)	626.8 (620.9, 632.6)	− 4.4 (− 12.7, 3.9)
Proportion (95%CI)			
Meeting MVPA guidelines (%) (≥ 60 min/day)	61.5 (57.3, 65.8)	36.7 (36.7, 40.7)	− 24.9 (− 30.3, − 19.4)**
Meeting sleep guidelines (%) (≥ 9 & ≤ 11 h sleep/night)	44.0 (39.6, 48.4)	48.4 (44.2, 52.6)	4.4 (− 1.6, 10.4)
Meeting screen guidelines ≥ 5 days (%) (< 2 h/day) (*n* = 1261)	67.9 (63.6, 72.1)	77.6 (74.0, 81.2)	9.8 (4.7, 14.8)**
Meeting screen guidelines ≥ 7 days (%) (< 2 h/day) (*n* = 1261)	53.0 (48.7, 57.2)	58.6 (54.9, 62.2)	5.6 (0.1, 11.1)*

Results of multi-level linear and logistic regressions estimating minutes per day, the proportion meeting guidelines for boys and girls, and the difference between boys and girls Regressions are adjusted for grade, LOTE, rurality, school type, ICSEA category and percentage of wear time, and clustering at school level

*< 0.05; ** < 0.01

We found 22% of boys and 16% of girls meet all 3 recommendations for MVPA, screen-time and sleep (Fig. 1).

### Association between sleep, physical activity, sedentary behaviors, and demographic characteristics overall and by gender

Higher grade was associated with less LPA minutes [**− **7.8 (95%CI, − 12.3, − 3.4) mins.day^−1)^] less minutes of MVPA [**− **6.5 (95%CI, − 8.8, − 4.2) mins.day^−1^], and more sedentary time [31.0 (95%CI, 22.7, 39.3) mins.day^−1^)]. Grade 6 students were less likely to meet the MVPA and sleep guidelines and reported a lower level of adherence to screen-time guidelines 5 days a week, compared to grade 4 students. Students who spoke a language other than English at home spent on average, 19.0 (95%CI, 3.7, 34.3) more minutes in sedentary activities, and had 21.0 min less sleep (95%CI − 32.5, − 9.8) compared to students who only spoke English at home. Students in small rural towns (MM5) had higher mean minutes of LPA [11.6, (95%CI, 3.5, 19.7)] and 1.98 (95%CI, 1.24, 3.18) times the odds of meeting screen time guidelines 5 days a week, compared to those attending schools in regional centers (MM2) (Table 3).

**Table 3:** Associations between accelerometry outcomes, meeting guidelines and demographic characteristics for all students

		Mean minutes per day (beta (95%CI))				Odds of meeting behavior guidelines (odds ratio (95%CI))		
**All**								
		**Light PA**	**MVPA**	**Sedentary**	**Total sleep**	**MVPA (≥ 60 min/day)**	**Sleep (9–11 h)**	**Screen (≤ 2 h/day) 1 to 5 + days/wk**
*Gender (ref male)*		14.0 (9.6, 18.4)**	− 13.5 (− 15.8, − 11.2)**	− 4.4 (− 12.7, 3.9)	*3.9 (− 2.4, 10.1)*	0.36 (0.28, 0.45)**	1.19 (0.94, 1.52)	1.66 (1.28, 2.16)**
*Year Level (ref grade 4)*		− 7.8 (− 12.2, − 3.4)**	− 6.5 (− 8.8, − 4.2)**	31.0 (22.7, 39.3)**	− 16.7 (− 22.9, − 10.4)**	0.60 (0.47, 0.76)**	0.48 (0.38, 0.61)**	0.65 (0.50, 0.84)**
*LOTE (ref: only English at home)*		0.9 (− 7.3, 9.1)	1.5 (− 2.8, 5.7)	19.0 (3.7, 34.3)**	− 21.0 (− 32.5, − 9.5)**	1.14 (0.73, 1.76)	0.49 (0.31, 0.78)**	*0.85 (0.54, 1.35)*
Rurality (ref: regional center (MM2))	*Large or medium rural town (MM3/4)*	6.4 (− 2.2, 15.1)	3.9 (− 0.7, 8.5)	− 4.7 (− 18.6, 9.3)	− 4.6 (− 14.4, 5.2)	1.50 (0.96, 2.32)	0.98 (0.67, 1.43)	1.46 (0.88, 2.41)
	*Small rural town (MM5)*	11.6 (3.5, 19.7)**	3.2 (− 1.1, 7.6)	− 11.8 (− 24.8, 1.3)	− 2.3 (− 11.5, 6.9)	1.37 (0.91, 2.07)	1.03 (0.72, 1.47)	1.98 (1.24, 3.18)**
*School type (ref: Gov)*		− 6.2 (− 16.9, 4.5)	− 4.0 (− 9.7, 1.7)	6.0. (− 11.8, 23.7)	3.9 (− 8.8, 16.7)	0.60 (0.34, 1.04)	1.28 (0.78, 2.09)	1.24 (0.63, 2.44)
*ICSEA (ref:* < *1000)*		− 2.1 (− 8.6, 4.3)	1.2 (− 2.2, 4.7)	− 1.5 (− 12.2, 9.2)	2.7 (− 4.9, 10.3)	1.23 (0.88, 1.71)	1.04 (0.77, 1.40)	1.42 (0.96, 2.11)

Results of multi-level linear and logistic regressions, adjusting for other outcomes, and percentage weartime, with school as random effects

*Ref* reference category, *MVPA* moderate to vigorous physical activity, *LOTE* language other than English, *MM* modified Monash, *ICSEA* index of index of community socio educational advantage

*p < 0.05; **p < 0.10

When stratified by gender, among boys, increasing grade-level was associated with less LPA [**− **11.3 (95%CI, − 17.3, − 5.3) mins.day^−1^], less MVPA [**− **6.3 (95%CI, − 10.0, − 2.7) mins.day^−1^] and more sedentary time [26.3 (95%CI, 13.7, 38.9) mins.day^−1^] (Table 4) With higher grade-level similarly seeing declines in MVPA, increased sedentary time and a reduction in sleep [**− **23.3 (95%CI, − 31.5, − 15.0) mins.day^−1^] among girls. Speaking another language other than English at home was associated with reduced sleep duration among boys [**− **18.1 (95%CI, − 34.6, − 1.6) mins.day^−1^] and girls [**− **22.0 (95%CI, − 38.2, − 5.9) mins.day^−1^]. Greater odds of meeting the self-reported screen-time recommendations 5 days a week were observed among girls attending schools with higher socioeconomic position [OR 2.18 (95%CI, 1.19, 3.99)] compared to schools with lower socioeconomic position. For boys, living in a large/medium regional town or small rural town, compared to a regional center, yielded higher odds of meeting the MVPA guidelines. No association with the MVPA guidelines among girls was observed for rurality, although higher odds of adherence to the screen-time recommendation was observed in girls attending schools in large/medium rural towns and small rural towns compared to those in regional centers.

**Table 4:** Associations between behaviors and demographic characteristics, for boys and girls

	Mean minutes per day (beta (95%CI))				Odds of meeting behavior guidelines (odds ratio (95%CI)		
	Light PA	MVPA	Sedentary	Total sleep	MVPA (≥ 60 min/day)	Sleep (9–11 h)	Screen (≤ 2 h/day) 1 to 5 days/wk
**BOYS**							
*Year level (ref grade 4)*	− 11.3 (− 17.3, − 5.3)**	− 6.3 (− 10.0, − 2.7)**	26.3 (13.7, 38.9)**	− 8.8 (− 18.3, 0.8)	0.65 (0.46, 0.92)**	0.61 (0.43, 0.87)**	0.67 (0.47, 0.96)*
*LOTE (ref: only English at home)*	− 0.1 (− 10.5, 10.3)	2.6 (− 3.7, 8.9)	15.0 (− 6.8, 36.8)	− 18.1 (− 34.6, − 1.6)*	1.03 (0.57, 1.87)	0.61 (0.33, 1.13)	0.65 (0.36, 1.18)
*MMM rurality (ref: regional center)*							
*Large or medium rural town (MM3/4)*	2.9 (− 8.9, 14.7)	6.4 (0.0, 12.8)*	− 7.0 (− 27.6, 13.6)	− 2.0 (− 17.7, 13.6)	2.25 (1.27, 3.98)**	1.04 (0.58, 1.85)	1.07 (0.60, 1.92)
*Small rural town (MM5)*	10.3 (− 0.4, 21.0)	3.9 (− 1.9, 9.6)	− 13.7 (− 32.1, 4.7)	1.1 (− 12.8, 15.0)	1.71 (1.03, 2.86)*	1.28 (0.77, 2.14)	1.28 (0.76, 2.16)
*School type (ref: Gov)*	− 9.7 (− 24.4, 5.0)	− 3.1 (− 11.2, 5.1)	5.9 (− 20.7, 32.5)	6.7 (− 13.4, 26.8)	0.79 (0.38, 1.66)	1.13 (0.54, 2.34)	1.47 (0.66, 3.28)
*ICSEA (ref:* < *1000)*	− 2.9 (− 11.3, 5.5)	2.3 (− 2.3, 6.9)	2.6 (− 12.2, 17.4)	− 2.3 (− 13.4, 8.9)	1.25 (0.82, 1.91)	0.90 (0.59, 1.36)	1.00 (0.66, 1.52)
**GIRLS**							
*Year level (ref grade 4)*	− 4.7 (− 11.2, 1.7)	− 6.2 (− 9.1, − 3.3)**	34.2 (23.2, 45.6)**	− 23.3 (− 31.5, − 15.0)**	0.58 (0.42, 0.80)**	0.38 (0.27, 0.53)**	0.67 (0.46, 0.98)*
*LOTE (ref: only English at home)*	1.2 (− 11.5, 13.8)	0.0 (− 5.6, 5.7)	21.0 (− 0.6, 42.6)	− 22.0 (− 38.2, − 5.9)**	1.26 (0.67, 2.34)	0.40 (0.20, 0.82)*	1.23 (0.57, 2.66)
*MMM rurality (ref: regional center)*							
*Large or medium rural town (MM3/4)*	7.6 (− 2.6, 17.9)	1.7 (− 2.8, 6.2)	− 2.5 (− 20.0, 15.0)	− 6.7 (− 19.2, 5.8)	1.07 (0.65, 1.75)	0.88 (0.53, 1.48)	1.96 (1.02, 3.79)*
*Small rural town (MM5)*	12.8 (2.9, 22.8)*	2.3 (− 2.1, 6.7)	− 7.8 (− 24.8, 9.2)	− 7.1 (− 19.3, 5.1)	1.05 (0.65, 1.71)	0.78 (0.47, 1.30)	2.84 (1.49, 5.41)**
*School type (ref: Gov)*	− 3.5 (− 16.7, 9.8)	− 4.6 (− 10.5, 1.2)	5.8 (− 16.8, 28.5)	1.8 (− 14.5, 18.2)	0.48 (0.24, 0.95)*	1.38 (0.69, 2.76)	0.97 (0.37, 2.56)
*ICSEA (ref:* < *1000)*	− 1.2 (− 10.3, 6.3)	0.8 (− 2.9, 4.5)	− 6.8 (− 21.0, 7.5)	8.3 (− 2.0, 18.6)	1.32 (0.88, 1.96)	1.23 (0.81, 1.88)	2.18 (1.19, 3.99)*

Results of multi-level linear and logistic regressions by gender, adjusting for other outcomes, and percentage weartime, with school as random effects

*Ref* reference category, *MVPA* moderate to vigorous physical activity, *LOTE* language other than English, *MM* modified Monash, *ICSEA* index of index of community socio educational advantage

*p < 0.05; **p < 0.10

Investigation of adherence to screen-time guidelines 7 days a week followed a similar pattern to the 5 days a week outcomes (Supp Table 2).

## Discussion

Our sample of rural and regional children in Victoria found that very few children (22% of boys, 16% of girls, 19% all) simultaneously met each of the current guidelines for sleep, screen time and MVPA. When we considered the guidelines separately, boys were engaged in more MVPA compared to girls, yet girls engaged in more LPA across the day. A third of girls (37%) met the guidelines for MVPA compared to 62% of boys, and overall adherence declined with increasing grade-level [OR 0.65 (95%CI 0.50, 0.84)]. Around half of children met the sleep guidelines (44% boys and 48% girls) and approximately three quarters met screen-time guidelines (68% boys and 78% girls). No differences were observed in device-measured movement behaviors by socioeconomic status, and living in a rural town (small/medium/large) was associated with increased MVPA (boys) and LPA (girls) compared to those in regional centers.

Tapia-Serrano and colleagues [9] conducted a meta review across studies in 23 countries finding overall adherence to the three aspects of the 24-h movement guidelines to be low, one in ten (10.3%), in primary aged children, with boys having higher adherence (11.1%) than girls (6.9%). Further the meta-analysis aligns with the gendered pattern of outcomes identified here reinforcing our finding that fewer girls than boys meeting all 3 movement guidelines. Programs promoting healthy movement behaviors in children should specifically target the synergies and unique drivers of boys’ and girls’ behavior.

It has long been recognized that gender differences in physical activity among children are likely to be primarily connected to different messages and expectations imposed on pre-pubertal girls and boys [45]. Despite efforts to address this gap (e.g., ‘This Girl Can’) [46], our findings are further evidence of the longstanding trend of girls participating in less MVPA than boys. This gender disparity is consistent across international [47], and within an Australian longitudinal study (2016) finding lower PA level in girls to be associated with influences at the school and family level, and lower extra-curricular sport participation [48]. In contrast to other literature [49], our results indicated that, while having higher levels of MVPA, boys also spent a greater amount of time in sedentary behaviors (measured by accelerometry) and were less likely to meet sedentary guidelines (self-report). A longitudinal Australian study of children in the transition years between primary and secondary school (approx. 10–14 years old) found a greater increase in sedentary time for boys compared to girls, primarily attributed to time spent on electronic games [50]. In the current study,

increasing grade-level was associated with declines in LPA, MVPA and increasing sedentary time in boys and declining MVPA, sleep and increasing sedentary time in girls. This finding is consistent with global evidence of an age-related decline in physical activity from childhood to adolescence [9].

Geographical location seemed to infer some benefit to levels of physical activity where children in rural towns were more active (MVPA boys, LPA girls), and met the self-reported screen time guidelines (overall and girls) than their counterparts in large regional centers. While accelerometers do not capture the context of engaged physical activity, this is in line with our expectation that children in rural areas spend more time outside [23], which is in turn associated with more physical activity [24]. While we did not have children from metropolitan areas in this sample, there appeared a gradient of increasing healthy behaviors with increasing rurality which is reflected in national self-reported data showing higher levels of adherence to PA guidelines in rural and remote areas compared to urban areas (30% compared to 20% respectively) [51], while the self-report Victorian Child Health and Wellbeing state-based survey found 53% of children in rural and remote areas met PA guidelines, compared to 51% in metropolitan areas in 2019 [52]. Future research into environmental influences on how children engage, access and utilize space is required to optimize the observed benefit in regional areas.

### Strengths

This study examines a previously under-reported cohort of children, notably regional and rural children in Victoria, Australia, prior to the COVID-19 pandemic [53]. This study employed a census-styled school recruitment protocol and high participatory (opt-out) student recruitment procedure, to provide new insights into a large, free-living sample in these communities. Wrist worn accelerometry is a strength compared to the majority of other studies that typically rely on self-report of movement behaviors. Australian [21] and international data [12] suggests self-report appears to overestimate guideline adherence compared with objective assessment, we have consistently observed regional Victorian primary school children in grade 4 and grade 6 under-report their MVPA compared to accelerometry [54, 55].

### Limitations

The geographical spread and low population numbers in regional and rural Australia meant we used the school ICSEA score to reflect socio-economic status [34] of children in preference to utilizing individual child postal address or the Australian Bureau of Statistics Socio-Economic Indexes for Areas indices [56]. This decision reflects that schools are central points of focus for children as in some regional Australian towns, only one postcode covers a large geographic area (e.g., Warrnambool) and ICSEA is more indicative of students’ socioeconomic position, as it is based on a combination of school factors (geographic location, proportion of indigenous students) and student factors (parents’ occupations, parents’ education) [34]. However, using this school level measure of socio-economic position may also result in misclassification of some students due to within school socio-economic heterogeneity which may mask the associations between extremes of socio-economic position and behavioral outcomes.

The device-based measurement of movement patterns represents a strength, yet data from 141 students were excluded due to issues with the quality or completeness potentially impacting observed findings. Additionally, we needed to employ a randomization procedure to device allocation, e.g., Class 6 A were invited to wear an accelerometer whereas 6B missed out. This was a necessity due to a limited number of available accelerometers, however, randomization of classes may have reduced this measurement bias. A further limitation was that children removed the accelerometer for contact sport, however, there was not a log system or diary to account for non-wear time, which may have resulted in under-reporting of physical activity time. Recreational screen-time data was deduced from self-report exposing results to understanding and interpretation of questions, social desirability and recall bias [57]. The data were collected shortly after the release of the updated 24-h movement guidelines, and while immediate impacts on practice or behavior may have been limited, it is important to consider this evolving context when interpreting findings. It is also acknowledged that accelerometer data handling decisions (e.g., cut-points, epoch lengths) significantly influence adherence estimates across studies and limit their generalizability [58].

Despite the large sample of regional and rural children, caution should be exercised in generalizing beyond regional and rural Victoria, which is characterized by wide variations in population density and small, medium and large population centers within several cases hundreds of kilometers of sparsely habited farmland between them. In other parts of the region multiple larger communities are co-located within 1 or 2 hundred kilometers centered around oceans or major trading centers. Regional and rural status is not uniform, and earlier show proximity to the coast impacted physical activity [59]. Cultural norms about active transport, television and other screen use will differ between locations. Finally, non-participation bias may have influenced the observed results whereby systematic differences exist between consenting and non-participating schools and students.

### Implications for practice and policy

Many countries have established 24-h movement guidelines informed by relationships between physical activity, health and wellbeing [1]. Our study of a unique Australian rural population largely mirrors the international picture and reinforces the same question as these other studies: What would ensure children are sufficiently active, reduce sitting and screen time, and get good quality sleep? Trials in the study show a whole-of-community intervention had impact on health-related quality of life and dietary behaviors, without measured effects on self-reported movement, though this study was severely interrupted by COVID-19 and associated lockdowns [60]. An earlier, similar trial in regional Victorian children also found improvements in health-related quality of life [61] and parallel non-significant changes in device-measured MVPA [62]. Studies are needed to unpack what intervention strategies work for whom within child populations, locations and settings. Such evidence should help deliver solutions tailored to the patterns observed here and elsewhere.

## Conclusion

We identified gender, age and geographic differences in observed movement patterns. This points out the drivers of these behaviors that are likely specific and different depending on local context. Efforts to improve movement patterns must be flexible and adaptive to local communities.

## Supplementary Information

Below is the link to the electronic supplementary material.

Supplementary Material 1 (DOCX 15.4 KB)

Supplementary Material 2 (DOCX 15.1 KB)

Supplementary Material 3 (PNG 15.1 KB)

Supplementary Material 4 (PNG 15.1 KB)

## Data Availability

No datasets were generated or analysed during the current study.
