# Supplementary material for: Adherence to 24-h movement guidelines among rural and regional children in Australia: an observational study
Source: Eur J Pediatr. 2025 Oct 6;184(11):659. doi: 10.1007/s00431-025-06444-7 (PMC12500778; doi:10.1007/s00431-025-06444-7)
Supplement: Supplementary file 1 — Supplementary Material 1 (DOCX 15.4 KB) [file 431_2025_6444_MOESM1_ESM.docx]

Supplementary Table 1: Modified Monash Model Categorisations

| Category | Name | Explanation |
| --- | --- | --- |
| MM1 | Metropolitan Area | Major cities – accounts for 70% of Australian population  ASCG – Remoteness Area 1 (Major Cities) |
| MM2* | Regional centres | ASGS-RA2 (Inner Regional) or RA3 (Outer Regional) areas that are in, or within 20km road distance of a town with population greater than 50,000 |
| MM3* | Large rural towns | ASGS-RA2 (Inner Regional) or RA3 (Outer Regional) areas that are not in MM2, and are in, or within 15km road distance of a town with population between 15,000 – 50,000 |
| MM4* | Medium rural towns | ASGS-RA2 (Inner Regional) or RA3 (Outer Regional) areas that are not in MM2 or MM3 and are in, or within 10km road distance of a town with population between 5,000 and 15,000 |
| MM5* | Small rural towns | All other areas within ASGS-RA2 (Inner Regional) or RA3 (Outer Regional) not covered in MM2, MM3 or MM4 |
| MM6 | Remote communities | ASGS-RA4 (Remote Areas) |
| MM7 | Very Remote communities | ASGS-RA5 (Very Remote Areas) |

ASCG: Australian Statistical Geography Standard; RA: remoteness area; MM: Modified Monash; *Classifications included in study sample

REFERENCE:

Australian Government, Department of Health, Disability and Ageing.Modified Monash Model. URL: [Modified Monash Model | Australian Government Department of Health and Aged Care](https://www.health.gov.au/topics/rural-health-workforce/classifications/mmm)

Australian Bureau of Statistics. Remoteness Areas, Australian Statistical Geography Standard (ASGS) Edition 3. URL: [Remoteness Areas | Australian Bureau of Statistics](https://www.abs.gov.au/statistics/standards/australian-statistical-geography-standard-asgs-edition-3/jul2021-jun2026/remoteness-structure/remoteness-areas)
