# Supplementary material for: Adherence to 24-h movement guidelines among rural and regional children in Australia: an observational study
Source: Eur J Pediatr. 2025 Oct 6;184(11):659. doi: 10.1007/s00431-025-06444-7 (PMC12500778; doi:10.1007/s00431-025-06444-7)
Supplement: Supplementary file 2 — Supplementary Material 2 (DOCX 15.1 KB) [file 431_2025_6444_MOESM2_ESM.docx]

Supplementary Table 2: Associations between meeting screen-time guidelines 7 days a week and demographic characteristics

|  | | **Odds of meeting screen guidelines (<=2hrs /day) - 7 days/wk**  **(Odds Ratio (95%CI))** | | |
| --- | --- | --- | --- | --- |
|  | | **All** | **Boys** | **Girls** |
| *Gender (ref male)* | | ***1.30 (1.03, 1.64)**** | n/a | n/a |
| *Year Level (ref grade 4)* | | ***0.72 (0.57, 0.91)***** | 0.85 (0.61, 1.20) | ***0.63 (0.46, 0.87)***** |
| *LOTE (ref: only English at home)* | | 0.89 (0.58, 1.36) | 0.76 (0.43, 1.36) | 1.08 (0.58, 2.03) |
| Rurality (ref: Regional centre (MM2) | *Large or Medium Rural town (MM3/4)* | ***1.76 (1.12, 2.75)**** | 1.80 (0.99, 3.25) | ***1.78 (1.10, 2.87)**** |
|  | *Small rural town (MM5)* | ***1.88 (1.24, 2.87)***** | ***1.81 (1.06, 3.08)**** | **1.86 (1.17, 2.98)**** |
| *School type (ref: Gov)* | | 0.88 (0.50, 1.53) | 1.35 (0.64, 2.85) | 0.59 (0.31, 1.13) |
| *ICSEA (ref: <1000)* | | ***1.41 (1.01, 1.97)**** | 0.86 (0.56, 1.31) | **2.38 (1.57, 3.60)**** |

Results of multi-level logistic regressions, adjusting for other outcomes, and percentage weartime, with school as random effects. Ref: reference category; LOTE: language other than English; MM: Modified Monash; ICSEA: Index of Index of Community Socio Educational Advantage; *p<0.05; **p<0.10
