# Supplementary figures and images for: Adherence to 24-h movement guidelines among rural and regional children in Australia: an observational study
Source: Eur J Pediatr. 2025 Oct 6;184(11):659. doi: 10.1007/s00431-025-06444-7 (PMC12500778; doi:10.1007/s00431-025-06444-7)

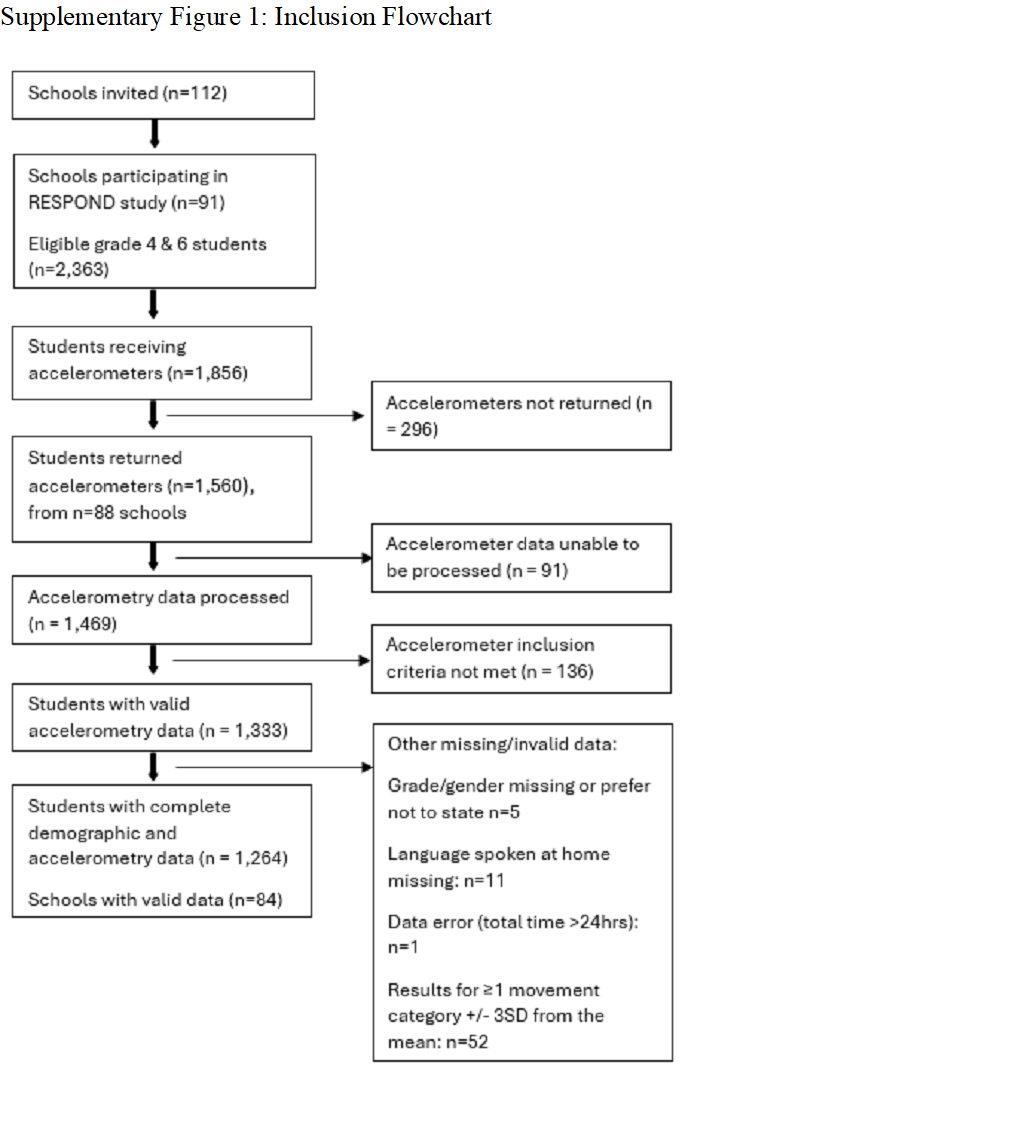

Supplement: Supplementary file 3 — Supplementary Material 3 (PNG 15.1 KB) [file 431_2025_6444_MOESM3_ESM.png]

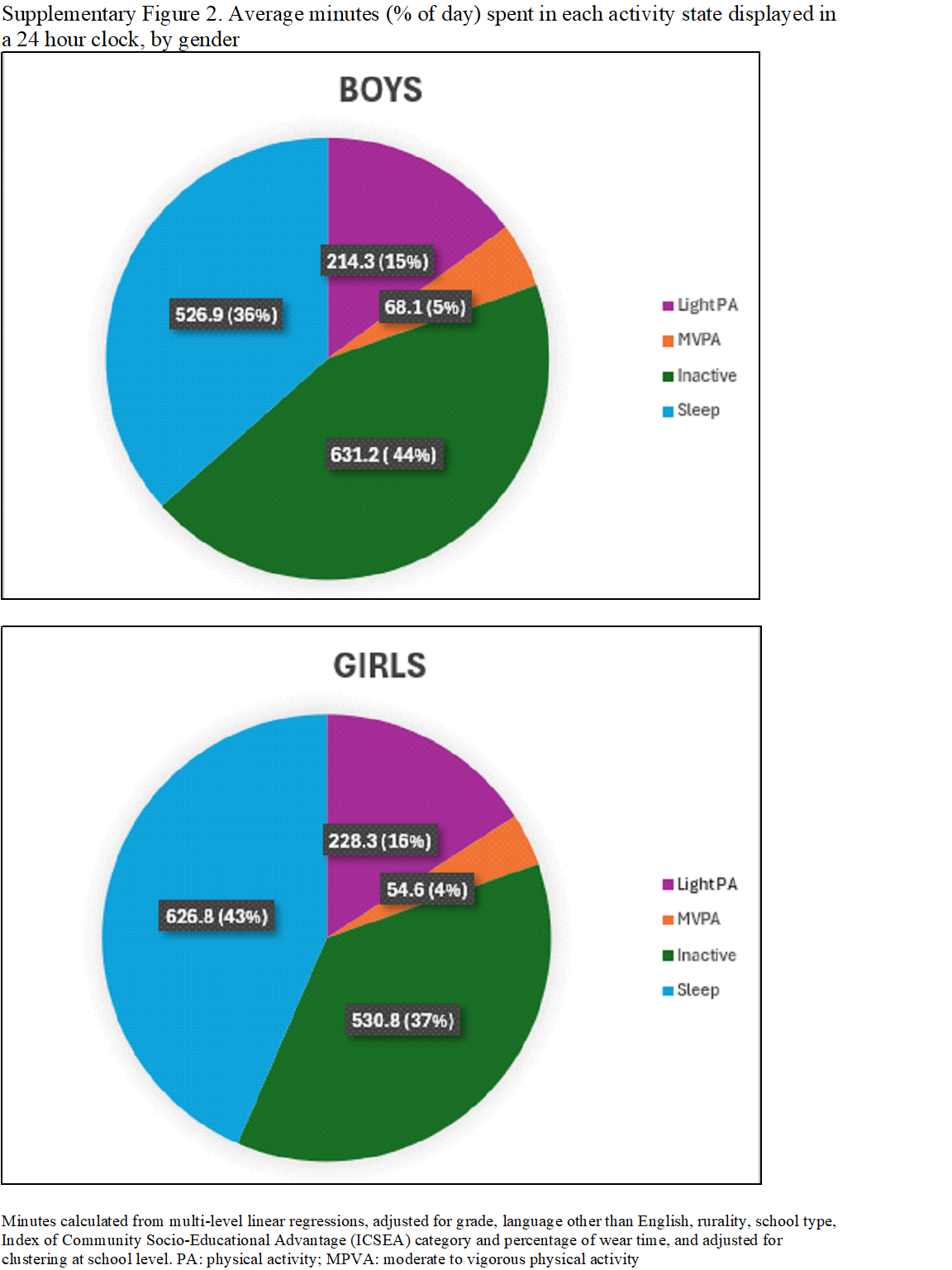

Supplement: Supplementary file 4 — Supplementary Material 4 (PNG 15.1 KB) [file 431_2025_6444_MOESM4_ESM.png]
